# 肿瘤代谢标志物在肺癌液体活检中的研究进展

**DOI:** 10.3779/j.issn.1009-3419.2023.106.29

**Published:** 2024-02-20

**Authors:** Shishi ZOU, Ning LI, Tianyu ZHANG, Qing GENG

**Affiliations:** 430060 武汉，武汉大学人民医院胸外科; Department of Thoracic Surgery, Renming Hospital of Wuhan University, Wuhan 430060, China

**Keywords:** 肺肿瘤, 代谢标志物, 液体活检, 代谢组学, Lung neoplasms, Metabolic biomarker, Liquid biopsy, Metabolomics

## Abstract

液体活检被逐渐应用于肺癌临床诊疗中。目前，随着代谢组学的发展，越来越多代谢标志物被认为是潜在的反映肿瘤发生发展的敏感标志物。本文概括了肺癌主要代谢途径的改变，包括葡萄糖代谢、氨基酸代谢、脂质代谢、鞘脂代谢、甘油磷脂代谢和嘌呤代谢。同时，本文综述了代谢标志物在肺癌早期诊断、预测疾病进展、评估化疗与免疫治疗疗效的作用，旨在为肿瘤诊疗提供有效的标志物。

## Abstract (en)

Liquid biopsy is gradually being applied in the clinical diagnosis and treatment of lung cancer. At present, with the development of metabolomics, more and more metabolic biomarkers are considered as potential sensitive markers reflecting the occurrence and development of tumors. This article summarizes the changes in the main metabolic pathways of lung cancer, including glucose metabolism, amino acid metabolism, lipid metabolism, sphingolipid metabolism, glycerophospholipid metabolism, and purine metabolism. Meanwhile, this article reviews the role of metabolic biomarkers in the early diagnosis of lung cancer, predicting disease progression, and evaluating the efficacy of chemotherapy and immunotherapy, aiming to provide effective biomarkers for tumor diagnosis and treatment.

肺癌是目前世界发病率第二位、死亡率第一位的癌症^[1]^。肺癌病死率降低的重要策略是早期筛查和诊断。液体活检是在肿瘤实质之外对血液等体液进行的一种微创活检。目前进行液体活检的有效生物标志物有循环肿瘤细胞、无细胞DNA、外泌体miRNAs（microRNAs）等，利用生物标志物的生物特性，可辅助疾病早期诊断，并对患者病情进展和治疗效果进行判断，从而达到精准治疗肿瘤的目的。代谢重编程是肺癌重要的表型变化，以适应肺癌能量供应及蛋白表达。代谢组学是继基因组学和蛋白质组学之后的一门新学科，主要研究包括碳水化合物、氨基酸、核苷酸、羧酸和脂类等在内的代谢底物和产物。代谢组的复杂性远低于基因组或蛋白质组，代谢物既是细胞的构建组成部分，也是参与细胞内信号传递的介质，最接近地反映了当前表型的状态，并告诉我们在生物体中到底发生了什么。目前，一些“经典”癌症生物标志物被提出用于肺癌检测。细胞角蛋白19片段（cytokeratin fraction 21-1, CYFRA 21-1）、癌胚抗原（carcinoembryonic antigen, CEA）和神经元特异性烯醇化酶（neuron-specific enolase, NSE）等通常在肺癌患者中的诊断敏感性为42%，特异性为83%^[2]^。因此，以代谢为中心的肺癌标志物在肺癌早期诊断、疾病分期、治疗效果、预后判断中具有极大的潜力^[3]^。

## 1 肺癌主要代谢途径变化

### 1.1 葡萄糖代谢

20世纪20年代，奥托·沃伯格观察到肿瘤细胞葡萄糖摄取大大增加，丙酮酸盐转化为乳酸，而不考虑氧气的可用性或稀缺性，这一过程被称为“Warburg效应”。研究^[4]^表明，糖酵解代谢途径过剩及代谢产物的累积抑制肿瘤细胞的凋亡，促进肿瘤增殖与迁移能力。3-磷酸甘油酯（3-phosphoglyceride, 3-PGA）通过结合磷酸甘油脱氢酶（phosphoglycerate dehydrogenase, PHGDH）桥接葡萄糖水平变化与p53蛋白介导的细胞凋亡。同时，肺癌细胞在葡萄糖耗竭时表现出弹性的能量适应机制，如线粒体磷酸烯醇丙酮酸羧激酶2（phosphoenolpyruvate carboxykinase 2, PCK2）介导的糖异生^[5]^、单羧酸转运蛋白1（monocarboxylate transporter-1, MCT1）介导的乳酸摄取及能量代谢^[6]^。

### 1.2 氨基酸代谢

氨基酸参与肺癌的能量代谢、生物合成、氧化还原平衡等代谢途径。生糖氨基酸为三羧酸循环提供燃料。研究^[7]^表明，与非肿瘤区域相比，非小细胞肺癌（non-small cell lung cancer, NSCLC）中肿瘤区域的生糖氨基酸显著增加，其中包括丙酮酸前体[丝氨酸（serine, Ser）、甘氨酸（glycine, Gly）、苏氨酸（threonine, Thr）、丙氨酸（alanine, Ala）和酪氨酸（tyrosine, Trp）]、α-酮戊二酸前体[谷氨酸（glutamic acid, Glu）、谷氨酰胺（glutamine, Gln）和脯氨酸（proline, Pro）]和琥珀酰辅酶A前体[缬氨酸（valine, Val）、异亮氨酸（isoleucine, Ile）和甲硫氨酸（methionine, Met）]。肺癌细胞Gln摄取及分解增加，Gln酶将Gln转化为Glu和氨，转化为糖代谢及核苷酸生物合成的底物。Gly作为单碳代谢的单碳源，促进癌症细胞的增殖。同时，谷胱甘肽合成过程中，Gly与Glu发挥重要作用，并维持氧化酶和抗氧化酶之间的稳态，是维持肿瘤的高间充质细胞状态的关键步骤^[8]^。

氨基酸对肿瘤细胞增殖与凋亡有介导作用。癌症患者血浆苯丙氨酸浓度升高，这可能与苯丙氨酸羟化酶活性失调有关^[9]^。苯丙氨酸是一种必需氨基酸，它不仅调节和控制物质代谢，而且通过Fas受体介导的细胞死亡受体途径、Rho/ROCK途径或颗粒酶B信号介导的凋亡途径介导细胞凋亡^[10]^。以聚苯丙氨酸为介质的纳米颗粒是靶向肺癌等肿瘤的良好药物载体^[11]^。氨基酸参与肿瘤微环境的免疫调节。吲哚胺2,3-双加氧酶（indoleamine-2,3-dioxygenase, IDO）是一种关键的色氨酸分解代谢限速酶，可将色氨酸代谢为犬尿氨酸。IDO的激活会损害抗原依赖性T细胞的激活，并导致免疫耐受和免疫逃逸，从而促进肿瘤的发展和转移^[12]^，可作为肺癌极具潜力的免疫治疗靶点。精氨酸不仅促进癌细胞生长增殖，同时促进T细胞受体表达及T细胞活化，具有抗肿瘤效应。NSCLC中癌细胞通过精氨酸琥珀酸合酶1（argininosuccinate synthase 1, ASS1）利用瓜氨酸合成精氨酸，多余的精氨酸被转移至肿瘤浸润淋巴细胞，作为抗肿瘤免疫的燃料^[13]^。

### 1.3 脂质代谢

脂质代谢能满足肿瘤分裂过程中对膜合成和能量产生的迫切需求，从而维持参与癌症细胞生长和转移过程。脂质的摄取和储存增加以及参与其代谢的蛋白质和酶的表达增强发生在各种癌症中。在NSCLC中表皮生长因子受体（epidermal growth factor receptor, EGFR）突变诱导脂肪生成，激活PI3K/AKT/mTOR通路，并抑制磷脂酸磷酸水解酶1入核。甾醇调节元件结合蛋白（sterol regulatory element-binding protein, SREBP）由此易位进入细胞核，并作为转录因子参与脂质代谢的不同基因的转录，如脂肪酸合成相关的AcCoA羧化酶（acetyl-CoA carboxylase, ACC）、三磷酸腺苷-柠檬酸裂解酶（adenosine triphosphate citrate lyase, ACLY）、脂肪酸合成酶（fatty acid synthase, FASN）、硬脂酰辅酶A去饱和酶-1（stearoyl-CoA desaturase-1, SCD1）以及胆固醇合成和摄取相关的HMG-CoA还原酶（3-hydroxy-3-methylglutaryl-CoA reductase, HMGCR）和低密度脂蛋白受体（low-density lipoprotein receptor, LDLR）^[14]^。脂肪酸在肺癌中含量增高，然而不同脂肪酸对肺癌发生发展中具有多样性。单不饱和脂肪酸和胆固醇通过调节细胞膜的稳定性和流动性，有助于EGFR信号的激活，同时EGFR直接磷酸化SCD1的第55位酪氨酸（Y55）位点，促进单不饱和脂肪酸产生^[15]^。ω-3多不饱和脂肪酸（omega-3 polyunsaturated fatty acid, ω-3 PUFA）是抗肿瘤免疫的重要营养调节剂。然而ω-6 PUFA可产生花生四烯酸，增加炎症及肿瘤发生。ω-6/ω-3比值高提示肺癌高风险。

### 1.4 鞘脂代谢

神经酰胺是鞘脂代谢的中心分子，可作为复杂鞘脂如鞘氨醇-1-磷酸、鞘磷脂、神经酰胺-1-磷酸和葡糖基神经酰胺的前体。鞘磷脂除在细胞膜中的结构作用外，对癌症细胞的生长、增殖、存活、迁移、侵袭和转移也很重要。癌症细胞中通过鞘磷脂合成酶和鞘磷脂酶催化使得神经酰胺/鞘磷脂平衡向鞘磷脂合成增加倾斜^[16]^。同时，神经酰胺合成酶基因、鞘氨醇激酶2、葡萄糖神经酰胺合酶等鞘脂代谢关键酶在肿瘤细胞中增加。具有高β-1,3-N-乙酰葡糖胺基转移酶5（beta 1,3-N-acetylglucosaminyltransferase 5, B3GNT5）或低半乳糖-3-氧磺基转移酶1（galactose-3-O-sulfotransferase 1, GAL3ST1）表达的NSCLC患者预后不良，B3GNT5调控的乳糖/新乳糖系列糖脂/硫酸酯鞘脂代谢平衡可作为确定癌症生长和进展中鞘脂代谢重编程的检查点^[17]^。鞘脂代谢和鞘脂信号通路的失调可能成为早期NSCLC的一种新兴治疗策略^[18]^。

### 1.5 甘油磷脂代谢

胆碱摄取和代谢的关键酶胆碱乙酰转移酶（choline acetyltransferase, ChAT）和胆碱激酶α（choline kinase α, ChoKα）在肺癌组织中的表达增加，通过^11^C-胆碱PET/CT成像能够可视化癌症病变^[19]^。人类NSCLC患者的血浆乙酰胆碱（acetylcholine, ACh）水平和肿瘤ChAT表达与EGFR-酪氨酸激酶抑制剂（EGFR-tyrosine kinase inhibitors, EGFR-TKIs）治疗的反应和无进展生存率相关，这与通过ACh毒蕈碱受体3依赖性的Wnt信号通路相关^[20]^。

### 1.6 嘌呤代谢

嘌呤是生物体内丰富的底物，是细胞增殖的关键原料，也是免疫调节的重要因素。黄嘌呤氧化还原酶（xanthine oxidoreductase, XOR）是一种关键的限速酶，通过将次黄嘌呤转化为黄嘌呤和将黄嘌呤转化成尿酸来控制嘌呤分解代谢的最后两步。它在催化过程中产生活性氧。XOR表达在不同癌组织中具有异质性，正常肺内XOR低表达，由于对嘌呤代谢的需求增加和炎症反应的激活，肺腺癌中XOR表达显著增加^[21]^。同时作为嘌呤代谢的终产物，血浆尿酸高水平与不良预后相关^[22]^。

## 2 肺癌代谢标志物的作用及进展

### 2.1 可作为肺癌早期诊断标志物

在肿瘤进展过程中，T细胞活化存在着有氧呼吸向有氧糖酵解的代谢转换。一项研究^[23]^通过测量外周血单核细胞细胞外环境的相对酸化水平，揭示免疫系统细胞的代谢活性谱，作为疾病状态的指标，并可排除慢性阻塞性肺部疾病的影响，在癌症I期，检测的特异性提高到94.0%，检测的敏感性提高到97.3%。

血浆游离氨基酸谱可评估肺癌风险，提高肺癌早期检出率^[24]^。癌细胞内可见多胺生物合成和摄取增加、分解代谢减少。Singhal等^[25]^通过识别和定量癌症患者和对照组中的血浆样本中的亚精胺/亚精胺N1-乙酰基转移酶-1（spermidine/spermine N1-acetyltransferase 1, SSAT1）介导的多胺通路，发现多胺代谢合成所需氨基酸精氨酸、鸟氨酸下降，代谢产物精胺、亚精胺及二乙酰精胺含量明显上升。Huang等^[26]^利用激光解吸/电离联合质谱（laser desorption ionization mass spectrometry, LDI-MS）分析血清代谢组学，确定了一个由7种代谢物和相关途径组成的生物标志物组，用以诊断早期肺腺癌，由组胺、半胱氨酸、脂肪酸（18:2）、尿嘧啶、尿酸、3-羟基吡啶酸（3-hydroxypicolinic acid, HPA）、吲哚丙烯酸（indoleacrylic acid, IA）组成，灵敏度为70%-90%，特异性为90%-93%。仅HPA、IA在肺癌患者中含量下降，其影响肺癌发生发展的潜在机制尚未阐明。基于上述质谱平台，血清代谢组学联合蛋白质肿瘤标志物及影像学建立三模态模型，更有效地诊断早期肺腺癌^[27]^。

肺癌患者中的胆碱代谢、鞘脂代谢与甘油磷脂代谢表现出早期的代谢紊乱。Wang等^[28]^基于液相色谱串联质谱的靶向脂质组学建立脂质代谢标志物组合，可作为预测肺癌的独立因素。与癌旁肺组织相比，肺癌组织中5种磷脂酰胆碱（phosphocholine, PC）16:0_18:1、PC 18:0_18:1、PC 18:0_18:2、PC 16:0_22:6和PC 16:0_18:2及1种甘油三酯（triglyceride, TG）16:0_18:1_18:1表达升高，3种溶血磷脂酰胆碱（lysophosphatidyl choline, LPC）16:0、LPC 18:0和LPC 20:4的表达降低，其特异性为95.65%，敏感性为90.70%，曲线下面积（area under the curve, AUC）为0.9843。基质辅助激光解吸/电离联合质谱成像进一步验证肺癌组织中的脂质原位表达。在I期NSCLC的血浆样本中，以LPC a C26:0、LPC a C26:1、PC aa C42:4、PC aa C34:4为代谢标志物组合，AUC为0.903^[29]^。除PC aa C34:4外，肺癌组代谢物均上升，这可能与磷脂酰胆碱的酰基链延伸过程有关，酰基链长度增加是肺鳞状细胞癌最常见的特征之一。另一项研究^[30]^利用全定量靶向质谱分析靶向138种代谢物，LPC 20:3、PC-ae C40:6在健康对照组和I/II期NSCLC之间分别显著上升与下调。除此之外，联合β-羟基丁酸、柠檬酸和富马酸的代谢物标志组合AUC可达0.898。一项针对女性非吸烟肺腺癌患者的研究^[31]^发现了一个由3种脂质组成的血清脂质生物标志物组，包括FA（20:4）、FA（22:0）和溶血磷脂酰乙醇胺（lyso-phosphatidylethanolamine, LPE）（20:4），AUC为0.99。其中，仅多不饱和脂肪FA（20:4）在肺癌患者中下降，这可能与ω-3 FA的抗肿瘤效应有关。一项研究^[32]^分析478例肺癌患者与370例良性肿瘤患者的血液中筛查47个氨基酸和肉碱指标，肉碱将长链脂肪酸带入线粒体进行氧化并转化为酰基肉碱，酰基肉毒碱谱可以反映脂肪酸代谢状况及肺癌等疾病的发展。鸟氨酸和棕榈基肉碱代谢生物标志物对早期肺癌的诊断具有重要意义（AUC为0.81，精确度为75.29%，灵敏度为74%）。

目前尚不清楚胆固醇代谢在肺癌发生时的作用。一项2006-2015年大型前瞻性动态队列研究^[33]^中，中国唐山市开滦队列分析984例肺癌及121,513例非肺癌患者，结果显示肺癌患者中表现出高敏C反应蛋白（high sensitivity C-reactive protein, hsCRP）升高（>3 mg/L）和低密度脂蛋白胆固醇（low-density lipoprotein cholesterol, LDL-C）降低（<120 mg/dL）。另一项研究^[34]^结合代谢组学与转录组学发现不论肺癌分期如何，油酸、胆固醇、肌醇在肺癌患者中下调。INPP5A、MAP2K2、CAMK1D、GABARAPL1和NDUFS4/8等相关调控基因主要通过Ca^2+^信号参与候选生物标志物相关的生物途径，其中绝大多数与肺癌增殖密切相关。其中，从膳食中负调节胆固醇摄入的钙/钙调节蛋白依赖性蛋白1D（calcium/calmodulin-dependent protein 1D, CAMK1D）与肺癌的增殖有密切关系。作为胆固醇的代谢产物，脱氧胆酸可在早期肺癌患者术后观察到明显上升，提示癌症与胆固醇代谢的潜在联系^[35]^。

### 2.2 可预测肺癌进展

肺腺癌的病理进展是逐步演变的。在进展为浸润性肺腺癌之前，正常肺组织从非典型腺瘤性增生（atypical adenomatous hyperplasia, AAH）到原位腺癌（adenocarcinoma in situ, AIS）、微小浸润性腺癌（minimally invasive adenocarcinoma, MIA），最后进展为浸润性腺癌（invasive adenocarcinoma, IAC）。与AAH的血清样本对比，AIS中可观察到天冬酰胺增加、胱氨酸减少，AIS+MIA组中可观察到胱氨酸和缬氨酸的减少，AIS+MIA+IAC组中可观察到3-氯酪氨酸和磷胆碱的上升、12:0-肉碱和Glu的降低。同时，通过对不同预后IAC患者进行代谢分型，发现胆汁酸代谢的失调与不良预后高度相关。胆汁酸代谢的失调主要体现在代谢产物胆汁酸、甘胆酸、牛黄脱氧胆酸和脱氧胆酸的进展性上升^[36]^。脂质代谢谱在早期及晚期肺癌中也会出现变化，一项研究^[37]^通过基于新型PbS/Au层状衬底的LDI-MS方法对健康对照组、早期肺癌组与晚期肺癌组进行脂质代谢谱分析，发现与对照组相比，早期肺癌组中花生四烯酸、N-棕榈酰亮氨酸、PC（26:0）和鞘磷脂（sphingomyelin, SM）（d18:1/14:0）下调，PC（36:4）、PC（38:3）、PC（38:4）、SM（d20:1/20:4）和SM（d17:1/24:1）上调；但在晚期肺癌组中脂质变化并没有扩大差异，而是趋向恢复，提示脂质变化可能具有适应性。

研究^[38]^显示，最初的原发性癌症（initial primary lung cancer, IPLC）幸存者发生第二次原发性癌症（second primary lung cancer, SPLC）的风险增加了4-6倍。波士顿肺癌研究^[38]^对82例SPLC和82例匹配的IPLC对照组的血清样本进行非靶向代谢组学研究。5-甲硫腺苷（5-methyl thiophene acryloyl, 5-MTA）和苯乙酰谷氨酰胺在SPLC病例中的水平比IPLC对照组增加了大约1.5倍，提示基于代谢组学的风险分层可能有助于区分SPLC风险。5-MTA是一种由多胺途径合成的含硫核苷，也是嘌呤和甲硫氨酸回收途径中的中间体，5-MTA在肝癌细胞中表达降低，5-MTA的回补可通过抑制DNA合成及鸟氨酸脱羧酶（ornithine decarboxylase, ODC）活性抑制肿瘤细胞的增殖^[39]^。苯乙酰谷氨酰胺是苯乙酸盐和Gln的二肽产物，天然存在于人类尿液中，是鉴别恶性肿瘤的良好代谢标志物^[40,41]^。磷脂酰乙醇胺（phosphatidylethanolamine, PE）38:5及PE 40:5可作为区别多原发性肺癌与肺内转移的敏感标志物。PE 38:5灵敏度为0.95、特异性为0.92，PE 40:5特异性为1、敏感性为0.90，在肺内转移患者中较多原发性肺癌患者升高^[42]^。

### 2.3 可预测EGFR-TKIs耐药性

代谢差异可能是NSCLC对EGFR-TKIs耐药的重要环节。耐药性细胞相对于糖酵解更偏向于氧化磷酸化，因此在合并二型糖尿病的肺癌患者中，二甲双胍联合奥希替尼可通过使细胞恢复糖酵解以减弱肿瘤细胞的药物抗性^[14,43]^。获得性EGFR-TKIs耐药性肺癌患者中的Gln依赖性是异质性的，耐药细胞系对细胞能量来源的依赖程度不同，与Gln代谢相关的代谢产物在抗性细胞中较高。针对Gln代谢途径的调节可能导致耐药性的逆转。CB-839对Gln代谢的抑制可作为获得性EGFR-TKIs耐药肺癌的治疗工具^[44]^。Azuma等^[45]^在NSCLC患者接受程序性细胞死亡受体-1（programmed cell death-1, PD-1）抑制剂治疗前后的血清样本中分析血浆游离氨基酸和色氨酸代谢物。由Gly、组氨酸、Thr、Ala、瓜氨酸、精氨酸、色氨酸等组成的游离氨基酸谱可预测较好预后。3-羟基犬尿氨酸、邻氨基苯甲酸、喹啉酸等色氨酸代谢物积累，表明减弱的抗肿瘤免疫并预测不良预后。同时，一项研究^[46]^在49例诊断EGFR基因突变并接受埃克替尼治疗的肺腺癌患者中，根据无进展生存期进行药物疗效分层，分析其代谢差异，LPC 16:1、LPC 22:5-1和LPE 18:2在耐药患者中减少，Cer 36:1-3、Cer 38:1-3、SM 36:1-2和SM 42:2在耐药患者中增多，可能与ARID1A、ARID1B、BCR和RBM10四个同时突变的基因表型相关联。

### 2.4 可预测化疗疗效

代谢组学可预测肺癌患者化疗的获益程度。基于芯片代谢细胞仪平台和荧光代谢探针，对32例IV期肺腺癌患者的胸腔积液中罕见的播散性肿瘤细胞进行代谢表型分析，发现糖酵解与线粒体呼吸的比值与患者化疗药物反应和预后呈正相关，是良好的预测因素。同时，研究^[47]^发现具有高糖酵解2-NBDG探针表达的细胞展现更多的间充质样特征，而具有高线粒体呼吸探针C12R表达的细胞表现上皮细胞特征。在高N/R比率患者中，一种上皮-间充质转化过程相关的酪氨酸激酶受体（tyrosine-protein kinase receptor, AXL）表达升高，针对AXL的药物靶点可以调节药物抗性。Tian等^[48]^针对354例使用一线培美曲塞加铂双联用药的肺腺癌患者，根据疗效和生存结果，分析筛选出由7种代谢产物组成的潜在生物标志物模型，包括尿苷、十二烷酰基肉碱、L-棕榈酰基肉碱、胆碱、二甲基甘氨酸、亚牛磺酸和烟酰胺，其中仅烟酰胺在疾病进展组下降，AUC为0.909。

## 3 总结与展望

肺癌代谢标志物在早期疾病诊断、疾病进展、疗效监测及预后预测中具有巨大潜力。但是目前仍有一些问题与挑战。首先，内源性代谢物具有相同的基本化学结构和交错的代谢途径网，因此代谢物的微小结构差异可能来自不同代谢通路改变，可预测不同的肺癌发生发展方向^[49]^。其次，检测和鉴定所有代谢物来全面覆盖代谢组仍然是一个挑战，需要应用不同的检测平台以及开发代谢组数据库。由于代谢组具有高度动态性，并且对各种内部和外部因素敏感，因此需要验证获得的信息以保证一致性和可重复性^[50]^。目前，通过代谢组学与基因组学、蛋白质组学结合，并通过整合分析获得代谢物表型改变背后的深层遗传信息，有利于进一步了解代谢物的作用。开发一种低成本、高通量、高准确率的代谢组学检测应用于肺癌临床诊疗中仍是极有希望的。

**Competing interests**

The authors declare no competing interests.
